# Crazy-Paving: A Computed Tomographic Finding of Coronavirus Disease 2019

**DOI:** 10.5811/cpcem.2020.5.47998

**Published:** 2020-05-18

**Authors:** Megan Gillespie, Patrick Flannery, Jessica A. Schumann, Nathan Dincher, Rebecca Mills, Argun Can

**Affiliations:** * Jefferson Health - Northeast, Department of Emergency Medicine, Philadelphia, Pennsylvania; † Jefferson Health - Northeast, Department of Critical Care, Philadelphia, Pennsylvania

**Keywords:** Coronavirus disease 2019, COVID-19, crazy-paving

## Abstract

**Introduction:**

Coronavirus disease 2019 (COVID-19) is caused by severe acute respiratory syndrome coronavirus 2.^1^ COVID-19 first occurred in Wuhan, China, in December 2019, and by March 2020 COVID-19 was declared a global pandemic.^1^

**Case Presentation:**

We describe a case of a 52-year-old female with past medical history of asthma, type 2 diabetes, and previous tobacco use who presented to the emergency department with dyspnea and was found to be positive for COVID-19. We discuss the computed tomographic finding of “crazy-paving” pattern in the patient’s lungs and the significance of this finding in COVID-19 patients.

**Discussion:**

Emergency providers need to be aware of the different imaging characteristics of various stages of COVID-19 to appropriately treat, isolate, and determine disposition of COVID-19 infected patients. Ground-glass opacities are the earliest and most common imaging finding for COVID-19.^2^–^4^ Crazy-paving pattern is defined as thickened interlobular septa and intralobular lines superimposed on diffuse ground-glass opacities and should be recognized by emergency providers as a radiographic finding of progressive COVID-19.^2^–^4^

## CASE PRESENTATION

**Image 1:**
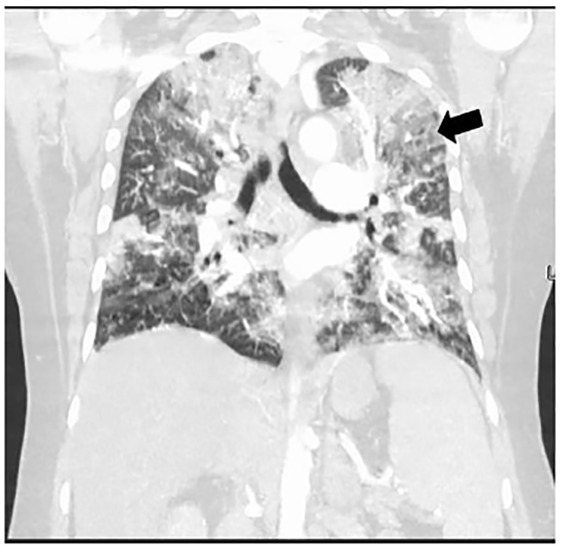
Crazy-paving pattern noted on computed tomography chest of coronavirus disease 2019 patient as manifested by multiple, patchy ground-glass opacities with reticular and interlobular septal thickening and intralobular lines in the coronal plane. Crazy-paving pattern can be seen in both lung fields, but the tile-like or stone pavement resemblance pattern is best noted in the left upper lung (arrow).

**Image 2:**
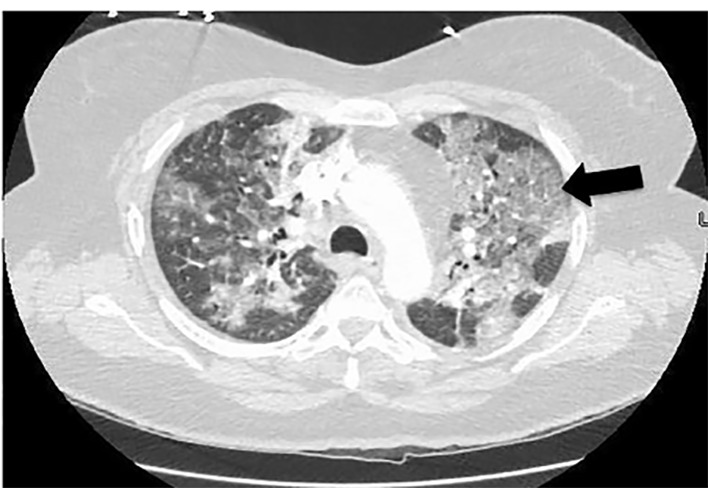
Crazy-paving pattern noted on computed tomography chest of coronavirus disease 2019 patient as manifested by multiple, patchy ground-glass opacities with reticular and interlobular septal thickening and intralobular lines in the axial plane. Crazy-paving pattern can be seen in both lung fields, but the tile-like or stone pavement resemblance pattern is best noted in the left lung (arrow).

**Image 3:**
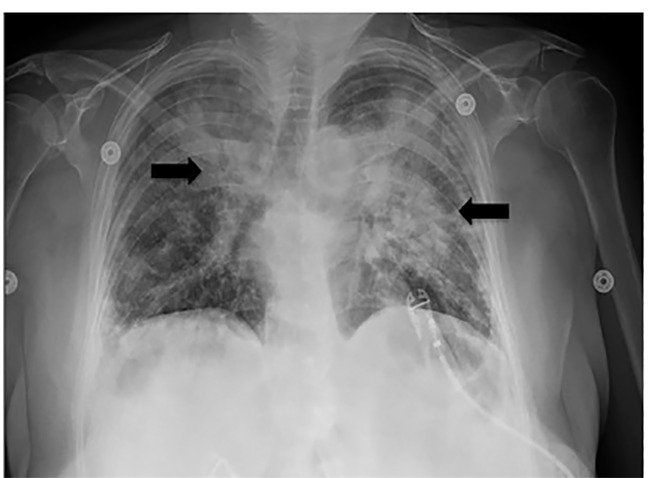
Radiograph of this patient with coronavirus disease 2019 demonstrates dense patchy airspace disease bilaterally (arrows).

A 52-year-old female with past medical history of asthma, type 2 diabetes, and previous tobacco use presented to the emergency department with dyspnea. The patient denied fever/chills, congestion, or gastrointestinal symptoms. She denied recent travel or exposure to known sick contacts. She presented afebrile, tachycardic, tachypneic, hypoxic with pulse oximetry measuring 79% on room air, and had mild conversational dyspnea with diminished auscultated breath sounds bilaterally. The patient had imaging findings as below (Images 1, 2, and 3) and laboratory abnormalities of elevated D-dimer, fibrinogen, lactate dehydrogenase, ferritin, C-reactive protein, lactic acid, glucose, aspartate aminotransferase, and alanine aminotransferase, in conjunction with a positive severe acute respiratory syndrome coronavirus 2 (SARS-CoV-2) reverse transcriptase polymerase chain reaction assay.

The patient was started on mid-flow supplemental nasal cannula oxygen at 15 liters per minute, enoxaparin, azithromycin, and ceftriaxone, and was admitted to the hospital.

## DISCUSSION

Coronavirus disease 2019 (COVID-19) is caused by SARS-CoV-2.^1^ The COVID-19 outbreak first occurred in Wuhan, China, in December 2019, and by March 2020, COVID-19 was declared a global pandemic.^1^ Emergency physicians are on the front line to diagnose and treat this global health emergency. These images are intended to present the “crazy-paving” pattern, which is a computed tomographic (CT) finding of progressive COVID-19.

**CPC-EM Capsule**

What do we already know about this clinical entity?

Ground-glass opacities are the most common and frequently noted radiographic abnormality of corona virus disease 2019 (COVID-19).

What is the major impact of the image(s)?

Crazy-paving pattern – thickened interlobular septa and intralobular lines superimposed on diffuse ground-glass attenuation – is an imaging finding suggestive of progressive COVID-19.

How might this improve emergency medicine practice?

Awareness of imaging findings of COVID-19 will help providers appropriately treat, isolate, and determine the disposition of infected patients promptly.

Ground-glass opacities, defined as hazy opacities compared to healthy lung, are the earliest and most commonly noted finding on CT for COVID-19.^2^–^4^ As COVID-19 progresses, a pattern known as “crazy-paving” can be noted on CT.^3^–^4^ Crazy-paving is defined by the Fleischner Society as thickened interlobular septa and intralobular lines superimposed on diffuse ground-glass attenuation, and is named for its resemblance to stone pavement streets.^2^–^5^ Crazy-paving pattern is classically noted as a finding of pulmonary alveolar proteinosis, a rare lung disease, but this pattern is also caused by *Pneumocystis jiroveci* pneumonia, sarcoidosis, bronchioloalveolar carcinoma, amiodarone-induced nonspecific interstitial pneumonia, lipoid pneumonia, organizing pneumonia, acute respiratory distress syndrome, pulmonary hemorrhage syndromes, and, now, COVID-19.^3^–^5^
